# What do we know about co-stimulatory and co-inhibitory immune checkpoint signals in ankylosing spondylitis?

**DOI:** 10.1093/cei/uxad032

**Published:** 2023-03-08

**Authors:** Christian Schütz, Xenofon Baraliakos

**Affiliations:** Rheumazentrum Ruhrgebiet Herne, Ruhr-University Bochum, Herne, Germany; Rheumazentrum Ruhrgebiet Herne, Ruhr-University Bochum, Herne, Germany

**Keywords:** spondyloarthritis, ankylosing spondylitis, co-stimulation, co-inhibition, co-signaling, immune checkpoints

## Abstract

Ankylosing spondylitis is the main entity of a family of inflammatory diseases affecting many musculoskeletal (sacroiliac joints, spine, and peripheral joints) and extra-musculoskeletal sites, termed spondyloarthritis. While it is debated whether disease onset is primarily driven by autoimmune or autoinflammatory processes, what is certain is that both innate and adaptive immune responses orchestrate local and systemic inflammation, which leads to chronic pain and immobility. Immune checkpoint signals are one key player in keeping the immune system in check and in balance, but their role in disease pathogenesis is still rather elusive. Therefore, we ran a MEDLINE search utilizing the PubMed platform for a variety of immune checkpoint signals in regard to ankylosing spondylitis. In this review, we summarize the experimental and genetic data available and evaluate the relevance of immune checkpoint signalling in the pathogenesis of ankylosing spondylitis. Markers such as PD-1 and CTLA-4 have been extensively studied and facilitate the concept of an impaired negative immune regulation in ankylosing spondylitis. Other markers are either neglected completely or insufficiently examined, and the data is conflicting. Still, some of those markers remain interesting targets to decipher the pathogenesis of ankylosing spondylitis and to develop new treatment strategies.

## Teaser

In this review, we summarize the experimental and genetic data available and evaluate the relevance of immune checkpoint signalling in the pathogenesis of ankylosing spondylitis. Markers such as PD-1 and CTLA-4 have been extensively studied and facilitate the concept of an impaired negative immune regulation in ankylosing spondylitis. Other markers are either neglected completely or insufficiently examined, and the data are conflicting. Still, some of those markers remain interesting targets to decipher the pathogenesis of ankylosing spondylitis and to develop new treatment strategies.

## Graphical Abstract

**Figure:**
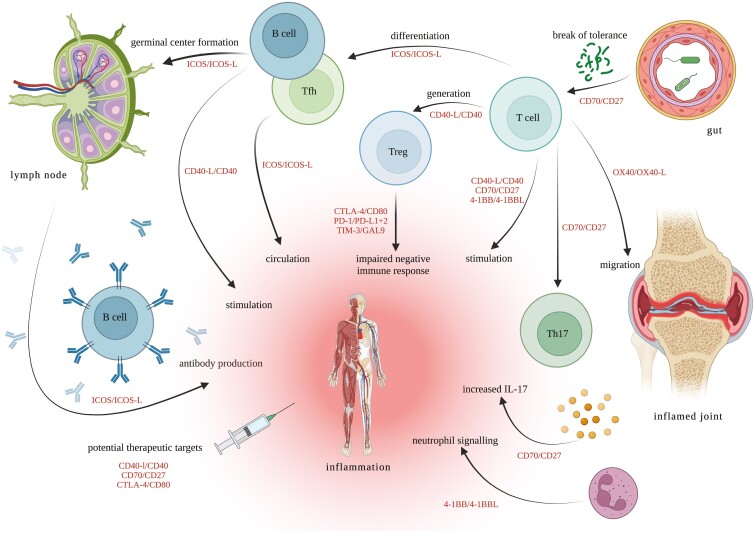

## Introduction

The term spondyloarthritis (SpA) represents different inflammatory clinical manifestations, such as inflammatory back pain, axial and peripheral arthritis, enthesitis, dactylitis, new bone formation or erosive axial, and peripheral joint disease. Furthermore, SpA emerges with the occurrence of extra-musculoskeletal manifestations such as uveitis, psoriasis, and inflammatory bowel disease [1]. In 2009 and 2011, the Assessment of Spondyloarthritis International Society (ASAS) categorized SpA into two main subgroups, the axial and the peripheral SpA (including PsA) [2]. The most prevalent type of SpA, ankylosing spondylitis (AS), also known as radiographic axial SpA, is a chronic inflammatory disorder. AS leads to bone remodelling and ankylosis mainly in the sacroiliac joints and the spine, which is consecutively associated with chronic pain and reduced mobility [3]. Despite its relatively frequent prevalence (0.3–1.4%) [4], there is limited knowledge about the exact pathogenesis of AS.

Several genetic markers have been associated with a high susceptibility for developing AS, with a prevalence for the human leukocyte antigen-B27* (HLA-B27*) [5]. Emerging evidence for a direct contribution of cytotoxic CD8^+^ T cells [6] and the detection of specific autoantibodies, such as anti-CD74 [7] suppose the involvement of an adaptive immune response in its pathogenesis. However, experimental data indicate the involvement of innate cells (mast cells, neutrophils, and macrophages), innate lymphoid cells (ILC), innate-like T cells [8], and the IL-23/IL-17 axis in the pathogenesis of AS [9], and to date biological disease-modifying anti-rheumatic drugs (bDMARDs), such as TNF-α and IL-17 blocking agents, are the major treatment options for AS [9]. This leads to the critical question of whether AS is an autoinflammatory or an autoimmune disease. Since AS may reflect characteristics from both types of immune responses, that is, unprovoked inflammation caused by activation of the innate immune system and a constant adaptive immune response to self-antigen involving B and T cells, this question cannot be conclusively answered. Mauro *et al*. have recently reviewed this topic and hypothesized a continuum between autoimmunity and autoinflammation, thereby favoring the view that onset of innate immune events precede the “disease-maintaining adaptive” immune processes [10].

Immune checkpoint signals (ICS) are regulators of the cellular immune system and are characterized as surface molecules that modulate intracellular signalling cascades positively (co-stimulatory) or negatively (co-inhibitory). Here we aim to gather the available data on co-stimulatory and co-inhibitory ICS in AS. Based on genetic and experimental data we summarize AS-specific ICS gene polymorphisms and delineate their immune cell or serum-specific expression profiles, respectively. This will help to recognize further fields of research related to the involvement of ICS in the pathogenesis of AS, possibly leading to new therapeutic intervention strategies.

## Search strategy and selection criteria

We ran a MEDLINE search utilizing the PubMed web portal (https://pubmed.ncbi.nlm.nih.gov) for original articles exclusively in English. We did not apply limits for the year of publication and used the following terms “spondyloarthritis” and “ankylosing spondylitis” in combination with “CD28, CD80, CD86, ICOS, ICOS-L, CD40L, CD40, OX40, OX40L, 4-1BB, 4-1BBL, CD27, PD-1, PD-L1, PD-L2, CTLA-4, TIM-3, B7-H3, B7-H4, FGL1, ICOS-L, CD160, BTLA, HVEM, TNFRSF14, CD70, B7-H7, B7-DC, GITR-L, GITR, OX40L, 4-1BBL, TIMG02, B7-DC, Galactin9, LIGHT”. We included articles on human primary material from patients suffering from AS and excluded articles on patients with other clinical SpA manifestations. Where clinical patient data were not provided or inconclusive, we made the assumption that the terms “ankylosing spondylitis” and/or “AS” indicated the exact clinical assessment criteria for AS (Table 1). Furthermore, we did not include animal data and data on killer-cell immunoglobulin receptors (KIR) and their role in SpA pathogenesis, since these have been reviewed elsewhere [11, 12].

**Table 1.** Publications on co-signaling molecules in AS summarized in this review regarding the immune checkpoint signals investigated. Molecules not displayed did not give any hit regarding applied search criteria. Numbers indicate references cited.

CD molecule	Synonym	Genetic data	Serum data	Expression data	Cell population data
CD27	–	–	–	[13]	[13–19]
CD28	–	[20]	–	[13]	[21–25]
CD40	–	–	–	[26, 27]	–
CD80	B7.1	–	–	[26, 28]	–
CD86	B7.2	–	–	[14, 26, 28]	–
CD134	OX40	–	–	[29]	–
CD137	4-1BB	–	–	[29]	–
CD137L	4-1BB ligand	–	–	[30]	[30]
CD152	CTLA-4	[31–37]	[37–40	[29]	
CD154	CD40 ligand	–	[41, 42	–	[43]
CD223	LAG3	[44]	–	–	–
CD276	B7-H3	[45, 46]	[45	[45]	–
CD278	ICOS	–	–	[13, 29]	[15, 16, 47, 48]
CD279	PD-1	[31, 49–51]	–	[29, 52–54]	[15, 47, 48, 54]
CD366	TIM-3	[55]	–	[52]	[56]
–	B7-H4	[46]	–	–	–

## Co-stimulatory signals in ankylosing spondylitis

### CD28–CD80 (B7.1)/CD86 (B7.2)

CD28 belongs to the CD28-superfamily and is a glycosylated, disulphide-linked homodimer cell surface protein of 44 kDa. CD28 is constitutively expressed on naïve T cells (Tn) of about 80% of CD4^+^ and 50% of CD8^+^ T cells. CD28 negative antigen-experienced T cells are considered to be reactivated independent of CD28 signaling and have been characterized as memory T cells (Tm). CD28 is the receptor for CD80 and CD86 and are both B7-family members. While CD86 is constitutively expressed on antigen-presenting cells (APC), CD80 is upregulated after toll-like receptor-dependent activation. CD28 shares both ligands with CTLA-4 which binds with a higher affinity and avidity [57]. Ligation of CD28 with its ligands is mandatory for Tn activation, survival, and cytokine production such as IL-2 and IL-6. Tn experiencing an antigen-specific signal without concomitant CD28:B7 signaling will become anergic [58].

Data on expression level and possible CD28 polymorphism in AS are very limited. Karakose Okyaltırık *et al*. investigated the relationship between CD28 polymorphisms and the occurrence of lung involvement in a Turkish AS patient cohort. While no significant differences between patients and healthy controls (HC) due to CD28 genotype expression could be detected, patients who did not have any CD28 T allele showed abnormal lung high-resolution computerized tomography scans, suggesting a possible association of CD28 variants with AS-related pulmonary involvement [20]. Furthermore, no significant difference in CD28 expression on total CD8^+^ or double negative mucosal-associated invariant T cells (MAIT) could be detected when comparing AS patients with HC [13]. Moreover, only studies discriminating effector (Teff) and Tm from other T-cell subpopulations by staining for CD28 have been conducted. Therein, both CD4^+^ and CD8^+^ CD28^−^ cells were found to be more expanded in AS patients than in HC [21–23]. CD4^+^CD28^−^ AS cells produced more perforin [22], INF-γ [22,24], TNF-α, and IL-10 [24] and showed lower amounts of spontaneous apoptosis[22] than their CD28^+^ counterparts. Furthermore, CD4^+^CD28^−^ cells lacked IL-2 and IL-4 expression [24], and presented TLR4 and to a smaller extent TLR2, whereas a correlation to HC could not be made [25]. One study demonstrated a direct relationship between the percentage of circulating cytotoxic CD8^+^CD28^−^ cells with a more severe course of disease [23]. Expression of the CD28 ligands CD80 and CD86 on monocyte-derived dendritic cells (mdDC) has been analyzed in active AS patients and HC. While CD80 expression was comparable on mdDC from both groups, CD86 tended to be expressed lower in AS patients but did not reach significance, nor for both CD80 and CD86 expression after stimulation of mdDC with lipopolysaccharide. These findings might be justified by a cohort size of only five patients [26]. Cantaert *et al*. provided evidence that only low levels of CD80 and CD86 are expressed on CD5^+^ B cells, whereas CD80 was expressed significantly lower on CD5^+^ than on CD5^−^ B cells. No variations could be detected between AS patients and HC. Interestingly, nonetheless, after B cell receptor (BCR) triggering upregulation of CD80 was only detectable at robust levels in HC, somewhat giving AS CD5^+^ HLA-DR^high^ B cells a more anergic phenotype [28]. Furthermore, no significant differences could be detected between CD86 levels on CD27^−^CD38^low^CD21^low^ B cells in AS and HC populations [14] (Table 2).

**Table 2.** Summarized ICS expression on and variations in immune cell subpopulations in AS. Only data generated from different AS groups or in comparison to healthy controls is displayed. Numbers indicate references cited.

Cell type	Subpopulation	Groups compared	AS specific findings
PBMC		AS to HC	No differences in PD1 expression [54]
Monocytes	CD14^+^	AS to HC	Increased B7-H3 expression [45]
DC	Monocyte derived	AS to HC	No differences in CD40 and CD80 expression [26]
B cells		AS to HC	No differences in expression [27]
	CD5^+^	AS to HC	increased frequency [28] Low levels of CD80 expression (compared to r-axSpA CD5- B cells) [28]
	CD27^-^CD38^low^CD21^low^	AS to HC	No differences in frequencies when co-expressing CD86 [14] Increased frequencies when co-expressing CXCR3 [14] Decreased frequencies when co-expressing T-bet and CD11c [14]
	CD19^+^CD27^-^	AS to HC	No differences in frequencies [16]
	CD19^+^CD27^-^CD95^+^	AS to HC	Increased frequency [18]
	CD19^+^CD27^dim^	AS to HC	No differences in frequencies [19]
	CD19^+^CD27^+^	AS to HC	No differences in frequencies [16] Increased frequency [18]
	CD19^+^CD27^high^	AS to HC	Increased frequency [16] Increased frequency only in patients with peripheral joint involvement [19]
	CD19^+^CD20^-^CD27^+^CD38^high^	Non-biologicals to biologicals AS	Decreased frequency [15]
	CD19^+^CD86^+^	AS to HC	Increased frequency [18]
T cells	CD3^+^	AS to HC	No differences in intracellular CTLA-4 expression [37]
	CD3^+^PD-1^+^	mSASSS >30 to <30	Decreased frequency [54]
	CD4^+^	AS to HC	no differences in ICOS [29] and PD-1 [52] expression Decreased OX40, 4-1BB [29] and TIM-3 [52] expression Increased PD-1 and CTLA-4 expression [29] No differences in intracellular CLTA-4 expression [37] Lower PD-1 expression after PHA-M stimulation [53]
	CD4^+^PD-1^+^	mSASSS >30 to <30	Decreased frequency [54]
	CD4^+^CD25^-/low/+/high^	AS to HC	Lower PD-1 expression after PHA-M stimulation [53]
	CD4^+^CD25^+^Foxp3^+^CD127^-^TIM-3+	AS to HC	Decreased frequency [56]
	CD4^+^CD28^-^	AS to HC	Increased frequencies [21–23]
	CD4^+^CXCR5^+^ICOS^+^	AS to HC	No differences in frequencies [47] Increased frequency correlated with disease activity [48]
	CD4^+^CXCR5^+^ICOS^+^	Non-biologicals to biologicals AS	Decreased frequency [15]
	CD4^+^CXCR5^+^ICOS^+^PD1^+^	AS to HC	No differences in frequencies [47]
	CD4^+^CXCR5^+^ICOS^+^PD1^+^	Non-biologicals to biologicals AS	Decreased frequency [15)
	CD4^+^CXCR5^+^PD1^+^	AS to HC	Increased frequency [16, 47]
	CD8^+^	AS to HC	Decreased PD-1 expression [52] No differences in PD-1 expression [53] No differences in intracellular CTLA-4 expression [37]
	Mucosal-associated invariant T cells (MAIT)	AS to HC	Decreased CD28 expression [13] No differences in CD27 and ICOS expression [13]

**Figure 1.**
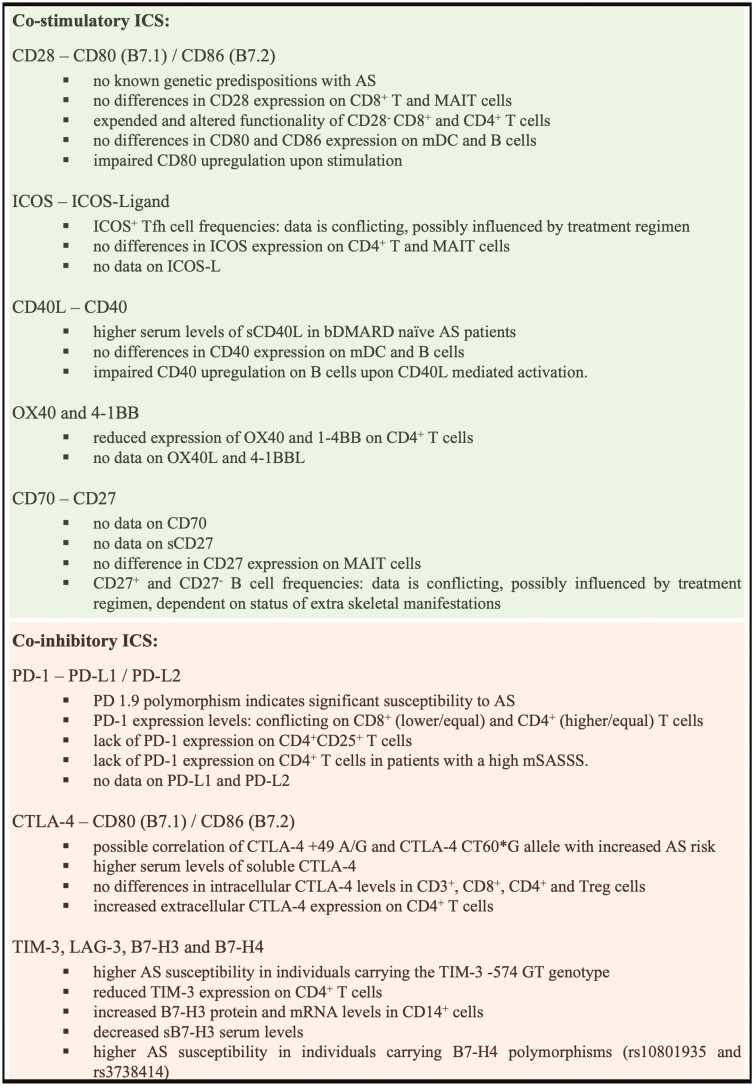
Take-home key facts on different ICS in AS pathogenesis. Bullet points displayed represent the most prominent findings discussed in the manuscript

In summary, direct signaling via the CD28/CD80/CD86 axis seems not to play an important part in AS pathogenesis, but a higher CD28^-^ frequency and impairment of CD80 upregulation might be a hint for an AS-specific functional dysregulation (Fig. 1).

### Inducible T-cell co-stimulator (ICOS)—ICOS-ligand

ICOS belongs to the CD28-superfamily and is a disulphide-linked homodimer cell surface protein of 55–60 kDa. The expression of ICOS is induced after TCR-crosslinking and/or CD28 co-stimulation. Therefore, ICOS is exclusively expressed on activated T cells and binds to its inducible T-lymphocyte co-stimulator ligand (ICOS-L). ICOS-L is expressed on APC and on epithelial and endothelial cells, especially under inflammatory conditions [59]. Hence, the biological function of ICOS-L is presumably very diverse. Ligation of ICOS with ICOS-L results in T-cell proliferation and cytokine production characterized by IL-4 [58].

Data on ICOS is mainly related to the investigation of follicular helper T cell (Tfh) cell subpopulations, and their functional capacity to activate B cells and is somewhat conflicting. While Xiao *et al*. demonstrated no differences in frequencies of both CD4^+^CXCR5^+^ICOS^+^ and CD4^+^CXCR5^+^ICOS^+^PD1^+^ cells isolated from peripheral blood of HC and drug naïve AS patients [47], Bautista-Caro *et al*. described a decrease of frequencies of the very same two T-cell populations of non-bDMARD-treated AS (AS/nb) patients compared to HC. Equal frequencies had been detected between bDMARD-treated AS patients and HC. Furthermore, co-cultures of CD4^+^CXCR5^+^ T cells with naïve CD19^+^CD27^-^ B cells demonstrated a decreased B cell helper capacity of Tfh cells. However, it is speculated that the cause is a decreased frequency of CD4^+^CXRC5^+^CXCR3^-^CCR6^+^ (Tfh-Th17) cells in AS/nb patients [15]. Other studies reported an increased frequency of CD4^+^CXCR5^+^ICOS^+^ Tfh cells in AS patients compared to HC [16,48], challenging the data from Xiao *et al*. [47]. Finally, ICOS expression levels on total, double negative and CD8^+^ MAIT cells did not vary significantly between AS patients and HC [13]. In a study investigating ICOS^+^ expression levels on CD4^+^ T cells, no differences have been reported [29]. As the evidence stands, there is no data available on ICOS-L expression in AS that might be related to its broad expression also on non-hematopoietic cells. This renders the interpretation of data from hematopoietic cell populations inconclusive.

In summary, the impact of ICOS/ICOS-L signaling in AS pathogenesis on the basis of the diverse data and with regard to its multiple roles in immunoregulation is not obvious [60]. Therefore, ICOS and ICOS-L remain interesting targets for further investigation of AS pathogenesis with focus on antibody production, circulation, and differentiation of Tfh and germinal center formation.

### CD40L–CD40

CD40L and CD40 are members of the tumor necrosis factor receptor super family (TNFRSF). CD40L is mainly expressed on activated T cells with utmost importance on Tfh cells, promoting B-cell activation and maturation by signaling through CD40, thereby ensuring class switch, memory B-cell generation and germinal center formation. Furthermore, CD40 is constitutively expressed in APC, such as DC and macrophages, and plays a pivotal role in their activation and related downstream immunological processes. Besides on T cells, CD40L is expressed on mast cells, macrophages, basophils, NK cells, B cells, and on non-hematopoietic cells, such as smooth muscle, endothelial, and epithelial cells, and is cleaved and shed from platelets into circulation through a MMP-dependent process (sCD40L) [61].

A study comparing platelet mRNA levels from AS patients and HC reported 4996 differentially expressed mRNAs, including many platelet-derived immune mediators, such as CD40L. Hence, CD40L might be one important factor triggering an inflammatory cascade and subsequent development of AS [43]. This assumption is supported by data from Stanek *et al*. reporting significantly higher levels of serum sCD40L in AS patients, bDMARD naïve, when compared to HC [41]. However, in another study no differences in serum sCD40L levels had been reported between AS and HC groups. Interestingly, about one-third of all patients received anti-TNF treatment, which might explain those study-related differences [42]. Surface expression of CD40 proved no differences on both mdDC [26] and B cells [27] between AS and HC groups. Yet, lipopolysaccharide-induced CD40 expression on mdDC was similar in both AS and HC groups [26]. The recombinant CD40L-mediated activation of B cells and subsequent expression of CD40 were significantly reduced in AS patients [27]. It can only be speculated if increased serum sCD40L levels are a countermeasure of the immune system to antagonize low CD40 expression on activated B cells or a result of missing binding partners.

In summary, the AS-specific amount of CD40L/CD40 data is very limited in the current research. Taking into account that therapy-sensitive elevated levels of sCD40L have been reported, several MMPs have been correlated to disease activity and progression and that CD40L/CD40 signaling plays multiple roles in T and B-cell stimulation and Treg generation renders this specific pathway of special interest for intervention strategies not only in AS [62].

### OX40 and 4-1BB

Both OX40 and 4-1BB molecules belong to the TNFRSF. OX40 is a secondary co-stimulatory signal that is expressed hours after full activation on T cells. OX40 ligand (OX40L) is stably expressed on APC and ligation to its receptor is responsible for the maintenance of an effective immune response that outlasts the first couple of days. OX40-OX40L engagement provides survival signals and enables differentiation of Tm. It also drives polarization toward a Th2 immune response, even in a low IL-4 environment [63]. Furthermore, there is data suggesting a possible role of OX40 in the migration process of T cells into inflamed synovial joints [64]. 4-1BB is a co-stimulatory ICS that is expressed on CD4^+^ and CD8^+^ activated T cell, DC, B cells, NK cells, neutrophils, and macrophages. 4-1BB binds 4-1BB ligand (4-1BBL) expressed on APC, thereby providing signals leading to enhancement of survival, proliferation, IL-2 secretion, and cytotoxicity of T cells [65].

Since there is no direct experimental or genetic data on OX40L and 4-1BBL, only one study has investigated the expression levels of OX40 and 4-1BB on CD4^+^ peripheral T cells. Frenz *et al*. reported a functional exhausted phenotype of CD4^+^ cells in AS patients along with reduced expression of OX40 and 4-1BB when compared to HC. This resulted in reduced IL-2 production and lower proliferative capacity after TCR-dependent stimulation. Furthermore, the authors pointed out that since the entire CD4^+^ T-cell compartment is exhausted in patients, higher infection rates are just as logical [29]. The only tentative indication of differentially regulated expression of OX40L in AS patients come from a study describing an AS-specific upregulation of the circular RNA (has_circ_0000652), simultaneously demonstrating increased OX40L expression in has_circ_0000652 treated THP-1 cells [30]. Whether or not this effect is reproducible in human primary macrophages is yet to be proven.

In summary, it remains elusive how much tribute can be paid to these two ICS pairs in AS pathogenesis. However, since OX40 involvement in T-cell migration in inflamed joints has been described [64] and that 4-1BB is known to be an important neutrophilic signaling molecule, as well as being a powerful T-cell stimulator [66], this may bring them more into the focus of future AS research.

### CD70–CD27

CD70 and CD27 are both members of the TNFSF. CD27 is constitutively expressed on naïve CD4^+^, CD8^+^ T cells on NK and hematopoietic stem cells. While downregulation of CD27 requires to prolong activation, it is not expressed on naïve but activated B cells. It can be shed from the surface of T cells by an MMP-dependent mechanism and binds to its only known ligand, CD70. CD70 is expressed on activated T, B, and NK cells, on macrophages and DC, and is induced by toll-like receptor, CD40, or antigen-receptor signaling. In T cells CD70–CD27 signaling shows characteristics of a co-stimulatory signal, resulting in enhanced survival, expansion, and Th1 polarisation, whereas in B cells CD27 signals are important for terminal differentiation. On the contrary, CD70 is suspected to dampen humoral immune responses. Interestingly, the CD70–CD27 pathway has also been described as immunosuppressive by counteracting Th17 cell activity and subsequent inhibition of IL-17 [67].

Data on CD27 elucidates T- [13, 17] and B- [14–16, 18, 19] cell frequencies and functional characteristics, thereby essentially utilizing CD27 for differentiation of subpopulations. Sugimoto *et al*. reported no difference in CD27 expression levels on MAIT cells from AS patients compared to HC [13]. Furthermore, a study comparing HLA-B27^−^ HC with a combined group of HLA-B27^+^ AS patients and HLA-B27^+^ HC demonstrated that CD8^+^CD27^+^CD45RA^-^ Tm and CD8^+^CD27^-^CD45RA^+^ Teff produced lower and CD8^+^CD27^+^CD45^+^ naïve and CD4^+^CD27^+/-^CD45^+/-^ T cells equal amounts of TNF-α and IFNγ after *in vitro* activation. Subsequently, the authors reasoned an HLA-B27* status-dependent influence on cytokine production of activated CD8^+^ T cells [17]. Data on B cells is extremely varied, arising in all probability from different patient cohort treatment regimens and varying subpopulation definitions with regard to the CD27^high/+/dim^ status. Studies on plasmablasts reported lower frequencies of CD19^+^CD20^-^CD27^+^CD38^high^ cells in AS/nb compared to AS/b [15] and higher frequencies for CD19^+^CD27^high^ cells in AS patients with peripheral joint involvement compared to either AS patients with axial involvement or HC [19]. Another study reported an increased frequency for CD19^+^CD27^high^ cells [16]. If these differences are due to differences in treatment regimen, completely naïve [16] to non-bDMARD [19], or other factors such as sex can only be speculated here. Another study reported significantly lower amounts of CD19^+^CD27^+^ memory B cells in AS patients than in HC and at the same time increased amounts of CD19^+^CD95^+^CD27^-^ and CD19^+^CD86^+^ B cells [18]. However, in a very recent study CD38^low^CD21^low^CD27^−^ B cells were reported to be elevated when co-expressing CXCR3 and decreased when co-expressing T-bet and CD11c in AS patients, an effect even more pronounced in patients with extra-skeletal manifestations [14]. In contrast, two studies showed no differences in naïve CD19^+^CD27^−^ and memory CD19^+^CD27^+/dim^ populations [16, 19].

In summary, even if not yet sufficiently examined, the CD70/CD27 signaling pathway represents an interesting target to be investigated in the context of AS. Besides the general involvement in T-cell activation, break of tolerance, and Th17 immune response modulation, evidence is generated from data in RA patients demonstrating an enhanced surface CD70 expression on CD4^+^ T cells in line with increased IL-17 and IFNγ production. If this pathway will hold its therapeutic potential, as discussed in several studies [67], further investigation is necessary.

## Co-inhibitory signals in ankylosing spondylitis

### PD-1-PD-L1/PD-L2

PD-1 is a surface marker of the CD28 superfamily and is expressed on activated T and B cells, NKT cells, monocytes, and DC. Its general role is the suppression of immune-inflammatory activity and prevention of autoimmune disease. PD-1 binds to PD-L1 (B7-H1) and PD-L2 (B7-DC). While PD-L1 is expressed on all hematopoietic and many non-hematopoietic cells, PD-L1 expression is inducible on macrophages, DC, and mast cells. After crosslinking of PD-1 with its ligand, suppression of inflammatory T-cell activity is mediated by induction of apoptosis in antigen-specific T cells and concomitant suppression of apoptosis in regulatory T cells (Treg). This comes along with reduced proliferation, cytokine production such as IL-2 and both initiation and retention of Foxp3 expression. Furthermore, PD-1 signaling in activated B cells leads to the inhibition of activation, proliferation, and survival [68].

Since PD-1 has a central immune regulatory function dampening inflammatory processes, many studies have investigated the PD-1 encoding gene PDCD1 polymorphisms and their association with AS susceptibility. The most widely studied polymorphisms are PD 1.3 G/A, PD 1.5 C/T, and PD 1.9 C/T. Meta-analysis focusing on at least those three polymorphisms revealed a significant contribution of the PD 1.9 polymorphism to AS susceptibility in Asian [49, 50] and overall [31, 50] but not in Caucasian populations [49]. However, the PD 1.3 polymorphism showed no correlation to AS susceptibility, and data on the PD 1.5 polymorphism is conflicting. Two meta-analyses revealed no significant association [31, 49], whereas one meta-analysis reported a significant association with AS susceptibility in Asian but not in overall populations [50]. However, since most polymorphism data has been generated from Asian cohorts, ethnicity-specific meta-analysis is still difficult to conduct. One recent study has reported that the frequencies of TT alleles in PD 1.9 polymorphism were significantly higher in acute anterior uveitis (AAU) diagnosed females without AS than in controls [51]. This might be of specific interest since, in AAU, AS is the most commonly associated disease and about 40% of AAU patients suffer from undiagnosed AS [69]. While there is no AS-specific expression data on PD-L1 and PD-L2, studies on PD-1 focus on T cells. Only two studies investigated PD-1 on CD8^+^ T cells, reporting opposing results. While Duan *et al*. showed that the percentage of PD-1 expression was lower in AS [52], Zhou *et al*. could not see any variations [53]. Furthermore, no differences in PD-1 expression levels have been reported in PBMC [54] and in unstimulated T cells [53]. However, Chen *et al*. could demonstrate that percentages of CD3^+^PD-1^+^ and CD4^+^PD-1^+^ cells were significantly lower in AS patients with higher modified Stoke Ankylosing Spondylitis Spinal Score (mSASSS) and reasoned that lack of PD-1 might facilitate radiographic progression [54]. While CD4^+^ cell ratio has been described to be higher in AS compared to HC [54], Frenz *et al*. identified an enhanced PD-1 expression on CD3^+^CD4^+^ cells in AS patients along with a general exhausted phenotype [29]. This contrasts with the findings of Duan *et al*., which showed no differences of PD-1 expression on CD4^+^ cells from AS and HC [52]. Interestingly, treatment with anti-PD-1 led to increased IL-2 production but was not correlated to PD-1 expression levels [29]. A study focusing on PHA-M stimulated T cells reported a significantly lower expression of PD-1 on CD4^+^CD25^high^ and CD4^+^CD25^+^ and to a lower extent on CD4^+^CD25^low^ and CD4^+^CD25^−^ cells, which may point to an AS-specific impairment of negative immune response regulation [53]. Data from studies on PD-1 expressing Tfh cells is in part conflicting, conducted in drug naïve [47], non-treated [16], or non-bDMARD [15] treated AS patients. Increased percentage of CD4^+^CXCR5^+^PD-1^+^ cells has been reported in two studies [16, 47] and was negatively correlated with the Bath Ankylosing Spondylitis Disease Activity Index (BASDAI) [47]. However, while one study reported no difference in percentages of CD4^+^CXCR5^+^ICOS^+^PD-1^+^ Tfh cells when compared to HC [47], another study reported lower frequencies [15].

In summary, since lack of PD-1 expression has been correlated to facilitate radiographic progression in mSASSS high AS patients and demonstrates a possible impairment of Treg cells to downregulate immune responses, PD-1 signaling is involved in AS pathogenesis. Still, there is no data on PD-L1 and PD-L2 available. Yet, it remains to be evaluated to what extent PD-1 signaling is causative for AS development.

### CTLA-4-CD80 (B7.1)/CD86 (B7.2)

CTLA-4 is a 33–37 kDa homodimer of the CD28 superfamily, which is exclusively expressed and highly regulated on T cell. In naïve T-cells, CTLA-4 is shuttled from intracellular vesicles to the surface after TCR and CD28-mediated activation, whereas Treg constitutively displays CTLA-4 on its surface along with steep-filled intracellular vesicles. Therefore, CTLA-4 expression in T cells is dependent on cell type and activation status. CTLA-4 competes with CD28 in binding CD80 and CD86 and is known to bind both with a much higher affinity and avidity [57]. The competitive nature of both molecules is even increased by the fact that, after binding CTLA-4 to either CD80 or CD86, these ligands are actively removed from the surface of APC by a process called trans-endocytosis. This process is part of the extrinsic signaling pathway. Intrinsic signaling pathways have been described mainly hypothesizing an inhibition of TCR/CD28 co-stimulation and an inhibition of immunological synapse formation, but data on both are still contradictory. Hence, CTLA-4 signaling downregulates T-cell activation and therefore is one key player in tolerance induction [58].

Multiple studies and meta-analysis have investigated the association of CTLA-4 polymorphisms with AS susceptibility, and gene chip analysis has recognized CTLA-4 is associated with higher AS risk [32]. No significant correlation could be detected for CTLA-4 + 49 A/G [31, 33–35] and CTLA-4 ^−^318 C/T [31, 35] polymorphisms focusing on overall populations. Only two studies reported a possible correlation of CTLA-4 + 49 A/G in an Iranian [33] and in a Caucasian [34] population, acknowledging that more research is necessary for verification. Another study could demonstrate a significant correlation between high AS risk in association with the CTLA-4 CT60*G allele, which was even more significant in females (>30 years) in an Algerian cohort [36]. Interestingly, it has been suggested that the CT60 short nucleotide polymorphisms located in the 3ʹ untranslated region influence soluble CTLA-4 (sCTLA-4) posttranscriptional modification and protein conformation [38], which may subsequently lead to altered functionality and quantity of sCTLA-4 [38,39]. Taking into account recently reported significantly higher sCTLA-4 serum levels in AS patients, one could speculate that a missing disease severity correlation could be due to CT60 polymorphism in some but not all of the patients investigated [40]. Further evidence may come from a study demonstrating significantly higher sCTLA-4 serum levels in AS correlating with BASDAI and C-reactive protein, simultaneously showing a preferential expression of posttranscriptional modified CTLA-4 mRNA levels in AS only. No changes in intracellular CTLA-4 levels could be detected in CD3^+^, CD8^+^, CD4^+^, and Treg cells [37]. Only one study reported an increased extracellular CTLA-4 expression on AS CD4^+^ cells when compared to HC [29].

**Figure 2.**
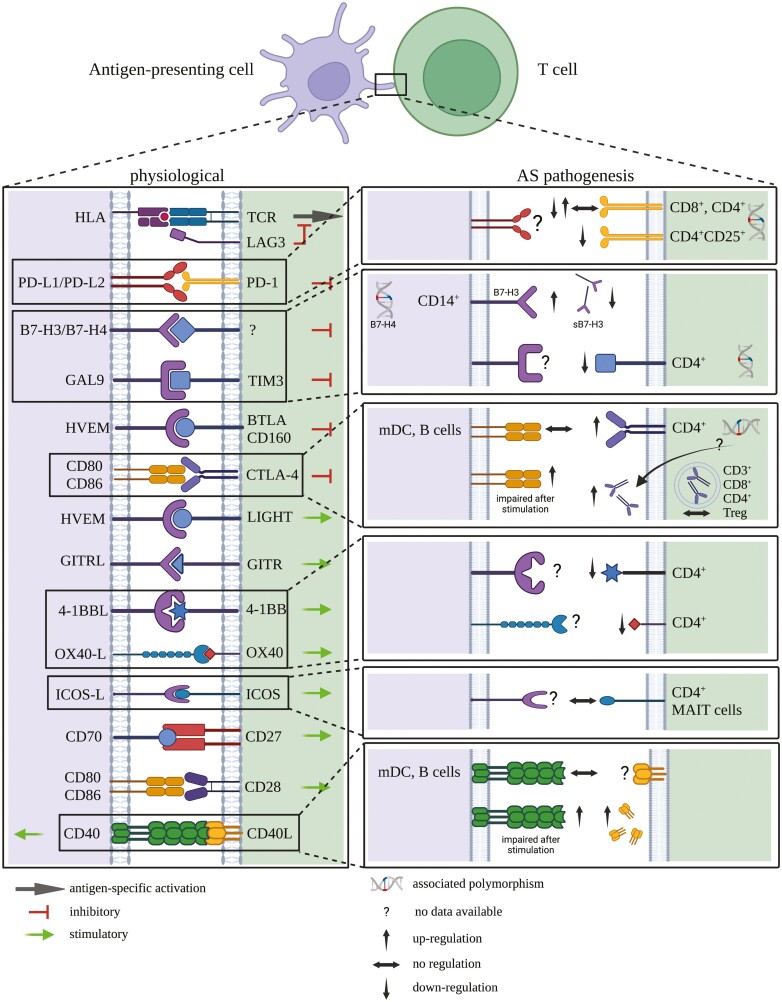
Schematic of immune checkpoint signaling under physiological conditions (left side) and in AS pathogenesis (right side). Only major findings that have been described for the most prominent signaling pairs are displayed. Fig. 1 provides a more detailed summary. Labeling on the left side indicates the corresponding receptor–ligand pairs. Labeling on the right side indicates the specific cell populations where variations have been reported

In summary, despite a limited number of studies, CTLA-4 dysregulation represents one component of AS pathogenesis, already discussed as a possible therapeutic target. Nonetheless, a pilot clinical study did not support this [70]. Interestingly, a CTLA-4 gene polymorphism has been associated with AS [36], possibly rendering sCTLA-4 unfunctional [38]. One might speculate that this contributes to an AS-specific ICS disbalance, but it remains to be further investigated to what extent and at which points in time CTLA-4 signaling is involved in the complex nature of AS pathogenesis (Fig. 2).

### TIM-3, LAG-3, B7-H3, and B7-H4

TIM-3 is a transmembrane molecule expressed on T cells and a variety of myeloid cells and binds to Galectin-9 (Gal-9). Together with other receptors, such as PD-1 and lymphocyte activation gene 3 (LAG-3), it is responsible for CD8^+^ T-cell exhaustion, resulting in reduced production of cytokines (TNFα, IFNγ, and IL-2) and proliferative capacity [71]. LAG-3 is a member of the immunoglobulin superfamily and inhibits proliferation, cytokine, and granzyme expression and facilitates Treg formation. It is expressed on conventional T cells, Treg, γδT cells, MAIT cells, B cells, invariant NKT cells, plasmacytoid DC, and neurons and has been reported to be essential for Treg suppressor function, T-cell homeostasis, and DC activation and maturation. The main ligands are HLA class II and fibrinogen-like protein 1 (FGL1) [72]. B7-H3 and B7-H4 both belong to the B7 family, including many ligands expressed on APC. B7-H3 is expressed on resting T and B cells and is upregulated on DC by IFNγ and on monocytes by GM-CSF signaling [58].

Wang *et al*. demonstrated an increased AS susceptibility in individuals carrying the TIM-3 ^−^574 GT genotype along with reduced mRNA and protein levels in CD4^+^, CD8^+^ T cells, and monocytes [55]. This is somewhat in line with reduced TIM-3 expression on CD4^+^ T cells from AS patients together with constrained IL-10 production and an overall reduced blood Treg population [52]. Furthermore, TIM-3^+^ cell frequencies in a CD4^+^CD25^+^Foxp3^+^CD127^−^ Treg population has been shown to be considerably reduced, together with significantly reduced expression of immune suppressive mediators such as IL-10 and TGF-ß in AS compared to HC [56]. Together this data indicates a disability of AS Treg to execute TIM-3-specific immune suppressive functions. Notably, a recent single-cell analysis of synovial AS Treg identified multiple Treg gene expression clusters, among those a Th-17-like Treg population expressing IL-10 and LAG-3. In *in vitro* autologous monocyte co-cultures, it was proven that LAG-3 expression on these Th-17-like-Treg directly inhibits IL-12/23 and TNF-α production, a finding with potential translational impact in AS pathogenesis [44]. Yan *et al*. investigated the expression profile of B7-H3 in AS patients and reported elevated protein and mRNA levels in CD14^+^ blood monocytes, whereas B7-H3 serum levels were significantly reduced compared to HC. Furthermore, B7-H3 serum levels negatively correlated with Ankylosing Spondylitis Disease Activity Score (ASDAS) [45]. Based on this finding, Chen et al. tested two B7-H3 (rs3816661 and rs3825859) and three B7-H4 (rs6428679, rs3738414, and rs10801935) polymorphisms regarding their correlation with AS susceptibility in a Chinese population. Interestingly, significant correlations could be found for two B7-H4 (rs10801935 and rs3738414) polymorphisms only [46].

In summary, the role of B7-H3 and B7-H4 in AS pathogenesis is not yet evident, even if the first studies have demonstrated dysregulation and higher polymorphism-specific AS susceptibility. While findings on a distinct Th17-like Treg-cell population seem to indicate a correct LAG-3 functionality, the combined data on TIM-3 rather strengthen the hypothesis of a general AS-specific impairment of negative immune regulation (Fig. 4). In any case, the collective data merit further investigation on the role of TIM-3, B7-H3, B7-H4, and LAG-3 in AS pathogenesis.

**Figure 3.**
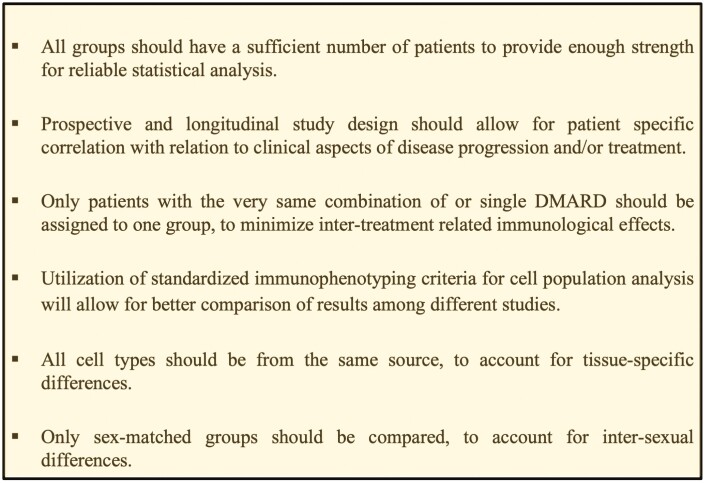
Factors to be considered in future study design to generate robust ICS data in AS pathogenesis. Bullet points are generated from limitations of studies summarized in this review. While some studies feature only one limitation, others combine different limitations.

**Figure 4.**
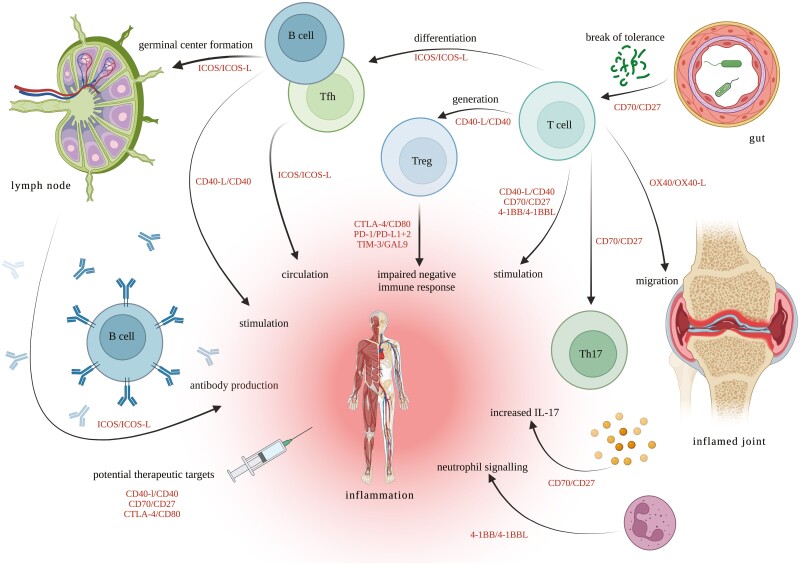
Immune checkpoint signals with possible impact on AS pathogenesis. The figure displays a selection of ICS pairs (in red) reviewed in the manuscript and their potential pathogenetic relevance in related immunological processes (in black) involved in AS development

## ICS targeting—implications for AS treatment

CD4^+^ and CD8^+^ T cells remain, besides others, major players in AS pathogenesis. This is partly related to the strong correlation of AS to HLA-B27* positivity and known T-cell reactivities against proteoglycan. To exert their immunological functions, T cells need proper activation, an antigen-specific signal, and a second signal, provided by stimulatory ICS. Otherwise, suppression of T cell responses and maintenance of self-tolerance needs signaling through inhibitory ICS [73]. These have been taken advantage of in cancer therapy, whereby checkpoint inhibitors (CPI), targeting inhibitory ICS, such as PD-1, PD-L1, or CTLA-4, were used to prevent cancer cells from evading the immune system [74, 75]. Interestingly, reports are mounting demonstrating emerging evidence that CPI treatment results in flares and outbreaks of immune-related adverse events (irAEs) in patients suffering from cancer. Thereby, irAEs affect any organ and even if the majority is low-grade and treatable, there are a decent number of high-grade events that might lead to permanent autoimmune disorders. Furthermore, the rate of flares of pre-existing autoimmune disorders in cancer patients varies greatly in range, between 25% and 50% for spondyloarthropathies after CPI therapy [76]. Interestingly, even if irAEs do not resemble all aspects of a de novo autoimmune disorder in non-cancer patients [77], the similarity is such that CPI-induced inflammatory arthritis has been recently discussed as a model to investigate early events in pathogenesis of autoimmune arthritis [78]. Mooradian *et al*. reported that of patients developing de novo rheumatic toxicities during cancer therapy, 84% had been treated with anti-PD1 only and 16% with anti-CTLA-4 alone or in combination with anti-PD1 [79]. Nigro *et al*. demonstrated evidence that anti-PD1 treatment accelerates PsA development in skin psoriatic patients, likely because anti-PD1 treatment leads to increased frequencies of Th1 and Th17 cells and subsequent enhanced production of IFN-γ, TNF-α, IL-6, IL-12, and IL-17 [80]. While anti-PD1 and anti-PD-L1 rescue mainly CD8^+^ T cells from their exhausted phenotype, anti-CTLA-4 results in activation of Teff (CD4^+^ and CD8^+^). Gremese *et al*. recently highlighted several major questions that emanate from the use of CPI in immune response modulation. One of the most intriguing questions focuses on AS: How to use CPI to treat autoimmunity [81]. So far, a first open-label prospective pilot study of 15 TNF inhibitor (TNFi) naïve (13% reached ASDAS40) and 15 TNFi non-responder AS patients (0% reached ASDAS40) treated with abatacept failed to reach endpoint [70]. This is of special interest since abatacept, a fusion protein of the extracellular domain of CTLA-4 and the Fc part of an IgG1 molecule, has proven effective in rheumatoid arthritis (RA) polyarticular juvenile idiopathic arthritis (P-JIA), and in psoriatic arthritis (PsA) not responsive to methotrexate (abatacept, EMA/CHMP/917551/2019, (31 January 2019)). The authors state a low sample size, only 6 months of treatment, and no dose comparison as possible limitations of the study [70]. In addition, the TNFi non-responder group was significantly older and displayed a higher disease duration. Collectively, this may indicate that ICS blockade works better during “early” disease. Together with missing sex distribution data, this might be of interest, since females get diagnosed later and TNFi treatment fails more often than in males. This might also indicate a sex-specific dependency on abatacept treatment efficacy [82]. Furthermore, the increased production of sCTLA-4 in AS compared to RA patients [37, 40] might interfere with a profound and rapid abatacept treatment efficacy in AS and may at least partly explain the differences seen in RA treatment settings.

## Discussion

Only 15 out of 36 examined ICS showed any results in our database search, demonstrating an ICS imbalance in AS pathogenesis rather than favoring activation of immune cells than inhibition. While much is known about ICS on T cells (Teff, Tm, Treg, Tfh, and MAIT) and B cells, only few publications did investigate other cell types such as DC and macrophages. Most of the T-cell-specific data is related to the CD4^+^ lineage, while data on CD8^+^ T cells is very limited. Only one study performed a combined analysis of multiple ICS [29], and data on T-cell subpopulations (Th1, Th2, Th17, Th9, Th22, Tc1, Tc2, and Tc17) and innate-like cells (ILC) respectively is missing. ILC3 is known to play a pivotal role in AS pathogenesis [83], and crosstalk with adaptive immune mechanisms by displaying inhibitory ICS among others has been described [84]. ILC do act tissue-specific [83] as well as anti-cytokine antibodies [85]. Therefore, tissue-specific phenotypes and functionality of immune cells should always be taken into consideration, together with other factors when designing studies (Fig. 3). In general, the plethora of distinct study designs limited the comparability and may be in part causative for conflicting ICS results. But those results may also be related to the fact that fine-tuning of ICS signaling is highly influenced by local environment-dependent immunological processes that differ over the course of the disease. The possibility that ICS signaling exclusively represents an epiphenomenon in AS pathogenesis may have to be discussed with respect to the course of the disease. At an initial disease stage, ICS dysregulation may facilitate break of tolerance, whereas at a later stage it may be mainly induced and functionally overwritten by long-time autoinflammatory processes. Otherwise, ICS expression is known to be modulated by environmental receptors and the resulting downstream signaling cascade [86], which may represent another kind of ICS involvement in AS pathogenesis. Regardless, combined therapeutic targeting of ICS remains a valid vision for AS treatment. Goenka *et al*. recently engineered a bifunctional co-stimulation inhibitor by covalently linking the extracellular domain of CTLA-4 with an anti-ICOSL antibody. Utilizing this construct, they demonstrated complete inhibition of CD80/86 binding to CD28 and ICOS binding to ICOSL [87]. Even if this approach is more likely a potential future treatment option for IgG-mediated inflammatory diseases than for AS, it highlights the potential of combined ICS-inhibitor approaches to handle autoinflammatory diseases. However, to better evaluate the exact potential of such an approach for AS treatment and the exact ICS combination necessary to achieve clinical efficacy supposedly in combination with other treatment strategies, further research is necessary to decipher the ICS-specific mode of action and pattern modulation in AS pathogenesis.

## Data Availability

Not applicable.

## Abbreviations

APC: antigen-presenting cells
AS: ankylosing spondylitis
ASDAS: AS Disease Activity Score
BASDAI: Bath Ankylosing Spondylitis Disease Activity Index
BCR: B cell receptor
CD: cluster of differentiation
CTLA-4: cytotoxic T-lymphocyte-associated protein 4
DC: dendritic cells
DMARD: disease modifying anti-rheumatic drug
ICOS: inducible T-lymphocyte co-stimulator
ICS: immune checkpoint signals
IFN-γ: interferon gamma
IL: interleukin
mdDC: monocyte-derived dendritic cells
MMP: matrix metalloproteinase
mSASSS: Stoke Ankylosing Spondylitis Spinal Score
PD-1: programmed cell death protein 1
PD-L1: programmed cell death protein ligand 1
PHA-M: phytohemagglutinin-M
RA: rheumatoid arthritis
SpA: spondyloarthritis
TCR: T-cell receptor
TLR: toll-like receptor
TNFRSF: tumor necrosis factor receptor superfamily
TNF-α: tumor necrosis factor alpha

## Permission to reproduce (for relevant content)

Granted within terms set out by the licensing agreement.
